# Palliative Gastrectomy versus Gastrojejunostomy for advanced Gastric cancer with outlet obstruction: a propensity score matching analysis

**DOI:** 10.1186/s12885-021-07904-7

**Published:** 2021-02-23

**Authors:** Xiao-Jiang Chen, Guo-Ming Chen, Yi-Cheng Wei, Hong Yu, Xi-Cheng Wang, Zhou-Kai Zhao, Tian-Qi Luo, Run-Cong Nie, Zhi-Wei Zhou

**Affiliations:** Department of Gastric Surgery, Sun Yat-sen University Cancer Center, No.651 Dongfeng Road East, Guangzhou, 510060 Guangdong China

**Keywords:** Advanced gastric cancer, Outlet obstruction, Gastrojejunostomy, Palliative gastrectomy, Overall survival

## Abstract

**Background:**

Gastric outlet obstruction (GOO) is a late complication of advanced gastric cancer, and it is controversial how to select the therapeutic strategies: gastrojejunostomy and palliative gastrectomy? Therefore, this study was to compare the surgical and survival outcomes of gastrojejunostomy and palliative gastrectomy.

**Methods:**

In total, 199 gastric cancer patients with outlet obstruction treated by surgery between January 2000 and December 2015 at Sun Yat-sen University Cancer Center were retrospectively reviewed. Patients were divided into gastrojejunostomy group and palliative gastrectomy group. Propensity score matching (PSM) was performed to balance the selection bias.

**Results:**

After 1:1 PSM, a total of 104 patients were included for final analysis. The median overall survival (OS) times in the gastrojejunostomy group and palliative gastrectomy group were 8.50 and 11.87 months, respectively (*P* = 0.243). The postoperative complication rates in the gastrojejunostomy group and palliative gastrectomy group were 19.23% (10/52) and 17.31% (9/52), respectively (*P* = 0.800), and no treatment-related death was observed. Multivariate analysis showed that periton0eal seeding (*P* = 0.014) and chemotherapy (*P* < 0.001) were independent prognostic factors. Among them, peritoneal seeding was a risk factor and postoperative chemotherapy was a protective factor.

**Conclusions:**

Our results indicated that although the surgical complications of palliative gastrectomy were manageable, it showed no survival benefit. Therefore, relieving obstruction symptom, improving patients’ quality of life and creating better conditions for chemotherapy appear to be the main therapeutic strategies for advanced gastric cancer with GOO.

## Introduction

Gastric cancer is responsible for over 1,000,000 new cases and an estimated 783,000 deaths in 2018, making it the fifth most frequently diagnosed cancer and the third leading cause of cancer death [1]. Various therapeutic strategies, including surgery, chemotherapy, targeted therapy, immunotherapy, and radiation therapy, have been demonstrated to improve the overall survival (OS) times for advanced gastric cancer [2].

Unfortunately, more than 80% of gastric cancer patients in China are diagnosed with advanced stages at the first clinic visit [3]. Gastric outlet obstruction (GOO) is a late complication of advanced gastric cancer. It leads to vomiting, inability to tolerate oral intake, weight loss, malnutrition for patients, which deprives them of the opportunity for proper treatment [4, 5]. Therefore, the treatment of GOO is of great importance for advanced gastric cancer with outlet obstruction.

There are currently three treatment options to alleviate GOO: endoscopic metallic stent insertion, gastrojejunostomy and palliative gastrectomy [5–7]. Among them, palliative gastrectomy is the radical therapeutic strategy that aimed to relieve the symptom and prolong the survival of the GOO patients. To date, few studies have evaluated the effect of palliative gastrectomy on those patients, the value of palliative surgical resection is still controversial. Although REGATTA trial demonstrated that the gastrectomy followed by chemotherapy could not show survival benefit compared with chemotherapy alone, its subgroup analysis indicated that the distal advanced gastric cancer may still benefit from gastrectomy [8]. Some previous retrospective studies have suggested that surgical resection may have a survival benefit [6, 9]. However, Okumura Y et al. reported that palliative gastrectomy offers no survival benefit as compared to gastrojejunostomy [10]. Up to now, most of the current studies were retrospective and have selection bias among enrolled patients.

Therefore, using the propensity score matching method to balance the selection bias, the aim of this study was to compare the safety and the impact on the overall survival of gastrojejunostomy and palliative gastrectomy for advanced gastric cancer with outlet obstruction.

## Materials and methods

### Patients

Patients were eligible if they were histologically proven diagnoses of gastric adenocarcinoma and outlet obstruction in Sun Yat-sen University Cancer Center between January 2000 and December 2015. All patients enrolled in the study were treated with surgery, including gastrojejunostomy and palliative gastrectomy. The palliative gastrectomy was performed by open surgery to remove the primary tumor and two-thirds of the distal stomach without dissection of the lymph nodes.

### Data collection

We reviewed the clinicopathologic characteristics before surgery and clinical outcomes after surgery of all patients. The characteristics included the age, gender, Eastern Cooperative Oncology Group performance score (ECOG PS), pre-procedure gastric outlet obstruction scoring system (GOOSS), baseline serum carcinoembryonic antigen (CEA), ascites, tumors around invasion of tissues and organs, degree of peritoneal seeding, distant organ metastasis, time of first oral eating after operation, average length of hospital stay, postoperative complications, postoperative chemotherapy, and overall survival. All the postoperative outcomes were enrolled in this study were occurred within 30 post-operative days.

GOOSS is defined as 0 for no oral intake, 1 for liquids only, 2 for soft solids, and 3 for low residue or full diet [11]. According to the first English edition of the Japanese classification of gastric carcinoma, the degree of peritoneal seeding` is classified as follows: P0, no peritoneal seeding; P1, disseminating metastasis to the region directly adjacent to the peritoneum of the stomach (above the transverse colon, including the greater omentum); P2, several scattered metastases to the distant peritoneum and ovarian metastasis alone; P3, numerous metastases to the distant peritoneum [12];

All regular follow-up assessments were completed by June 2019, and the median follow-up time was 15.31 months (range, 1.62–102.00 months).

This study complied with the Declaration of Helsinki and was approved by the institutional review board of Sun Yat-sen University Cancer Center. Informed consent was obtained from all individual participants included in the study.

### Propensity score matching Agesnalysis

The propensity score, defined as the conditional probability of receiving treatment, is commonly built in observational studies to adjust for selection bias [13, 14]. Because patients were not randomly assigned to the gastrojejunostomy and palliative gastrectomy groups, propensity score matching was applied to control the selection bias. In this study, the 1:1 nearest neighbor matching were chosen for the propensity score. Propensity score matching was performed using IBM SPSS version 22.0 (IBM SPSS, Armonk, NY).

### Statistical analysis

All of the statistical analyses were performed using the IBM SPSS version 22.0 (IBM SPSS, Armonk, NY). Chi square tests were used to compare categorical variables, and non-parametric tests were used to compare continuous variables. Univariate and multivariate analyses for survival were performed using Cox’s regression analysis. Variables with a *p* value < 0.05 in the univariate analysis were entered into multivariate analysis using Cox proportional hazard regression models. The forward selection method was used for multivariate Cox proportional analysis. The hazard ratio (HR) and 95% confidence interval (CI) were used to estimate the survival predictor.

The overall survival (OS) was calculated from the diagnosis of gastric outlet obstruction to death. Kaplan-Meier survival curves with log-rank testing were performed to compare the survival benefits. Two-sided *P* values< 0.05 were considered to be significant.

## Results

### Patient baseline characteristics

In this study, a total of 199 patients were included, with 89 patients in the gastrojejunostomy group and 110 patients in the palliative gastrectomy group. Table 1 shows the baseline characteristics of the 199 patients. As shown in Table 1, compared with patients in the palliative gastrectomy group, patients in the gastrojejunostomy group had poorer PS (*P* = 0.028), higher CEA (*P* < 0.042), more ascites (*P* = 0.003), more invasion of adjacent organs (*P* < 0.001), serious peritoneal seeding (*P* = 0.016), less periods of chemotherapy (P < 0.001), indicating the obvious selection biases between these two treatment strategies. Therefore, the covariates for propensity score matching were PS, CEA, ascites, invasion of adjacent organs, peritoneal seeding, periods of chemotherapy. After 1:1 PSM, the covariates were balanced, with both of 52 patients enrolled in the gastrojejunostomy group and palliative gastrectomy group, respectively (Table 1).

**Table 1:** Clinicopathological characteristics of gastric cancer patients with outlet obstruction before and after propensity score matching

Characteristics	Before propensity score matching		*p* value	After propensity score matching		*p* value
	Gastrojejunostomy group (%)	Palliative gastrectomy group (%)		Gastrojejunostomy group (%)	Palliative gastrectomy group (%)	
No. of patients	89	110		52	52	
Age (years)			0.701			0.694
≤60	51(57.3)	66(60.0)		27(51.9)	29(55.8)	
> 60	38(42.7)	44(40.0)		25(48.1)	23(44.2)	
Gender			0.171			0.083
Male	67(75.3)	73(66.4)		33(63.5)	41(78.8)	
Female	22(24.7)	37(33.6)		19(36.5)	11(21.2)	
Body mass index (mean, range)	21.0 (13.1–31.9)	20.4 (14.2–31.4)	0.146	21.1 (15.6–28.5)	20.1 (14.2–27.3)	0.082
PS			**0.028**			0.527
0/1	58(65.2)	87(79.1)		34(65.4)	37(71.2)	
2/3	31(34.8)	23(20.9)		18(34.6)	15(28.8)	
GOOSS			0.325			0.823
0	30(33.7)	30(27.3)		13(25.0)	14(26.9)	
1	59(66.3)	80(72.7)		39(75.0)	38(73.1)	
CEA (ng/ml, mean, range)	3.1 (0.1–615)	2.4 (0.2–275)	**0.042**	4.3 (0.6–612)	2.3 (0.2–194)	0.103
Ascites			**0.003**			0.684
No	50(56.2)	84(76.4)		32(61.5)	34(65.4)	
Yes	39(43.8)	26(23.6)		20(38.5)	18(34.6)	
Invasion of adjacent organs			**< 0.001**			1.000
No	14(15.7)	61(55.5)		14(26.9)	14(26.9)	
Yes	75(84.3)	49(44.5)		38(73.1)	38(73.1)	
Peritoneal seeding			**0.016**			0.139
P0	12(13.5)	25(22.7)		7(13.5)	9(17.3)	
P1/2	32(35.9)	51(46.4)		17(32.7)	25(48.1)	
P3	45(50.6)	34(30.9)		28(53.8)	18(34.6)	
Period of chemotherapy			**< 0.001**			0.095
0	55(61.8)	33(30.0)		27(51.9)	21(40.4)	
1–4	16(18.0)	30(27.3)		10(19.2)	20(38.5)	
≥5	18(20.2)	47(42.7)		15(28.9)	11(21.1)	

### Survival

**Fig. 1:**
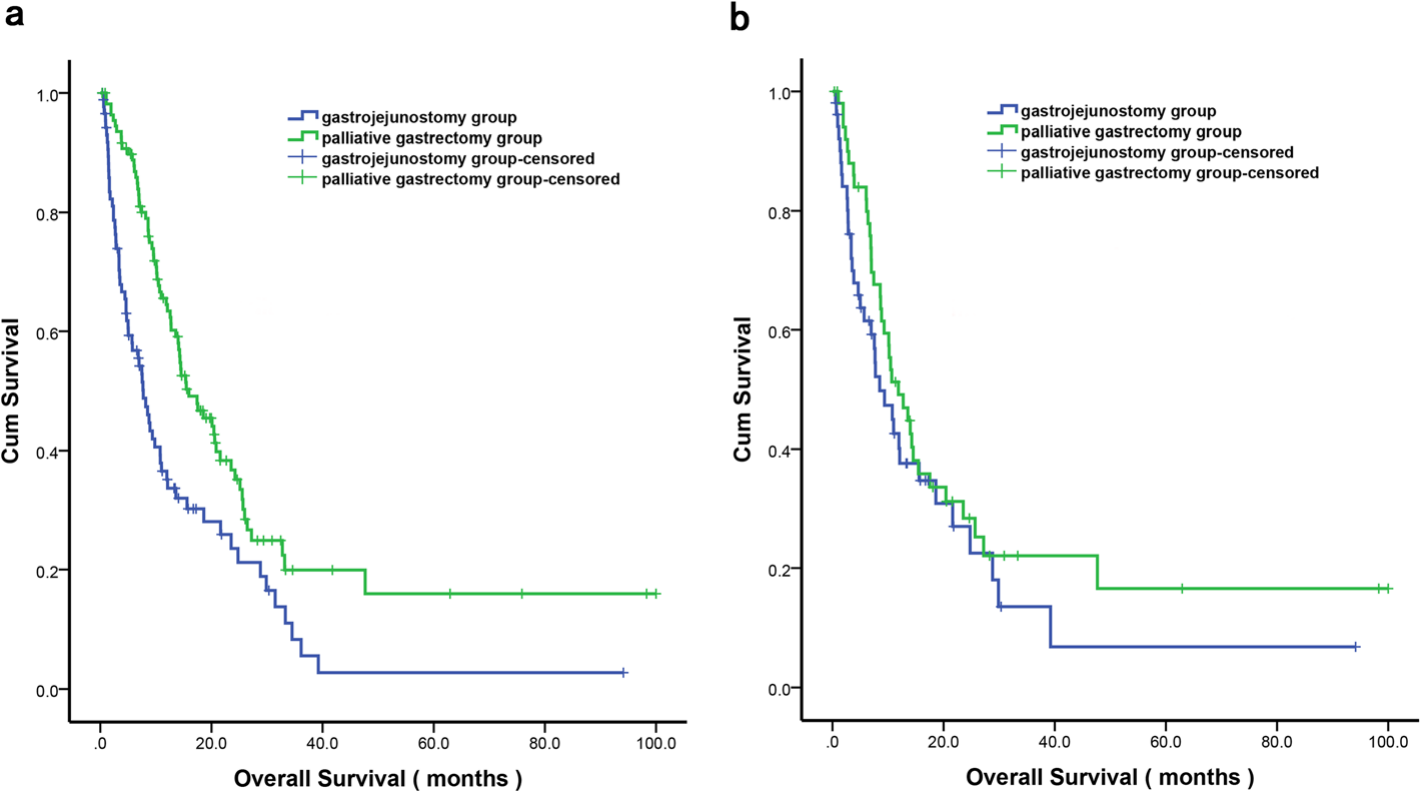
Kaplan-Meier survival curves of the palliative gastrectomy and gastrojejunostomy groups for gastric cancer patients with outlet obstruction (**a**) before propensity score matching (*P* < 0.001) and (**b**) after propensity score matching (*P* = 0.243). *P*-values were calculated using the log-rank test

Before PSM, the median OS was 7.73 (95%CI: 5.78–9.69) months in the gastrojejunostomy group and 15.93 (95%CI: 10.94–20.93) months in the palliative gastrectomy group, the OS difference between two groups was significant (*P* < 0.001) (Fig. 1 a). Nonetheless, after PSM, the OS difference was not significant (median OS: 8.50 (95%CI: 4.57–12.43) months vs 11.87 (95%CI, 7.37–16.36) month, *P* = 0.243) (Fig. 1 b).

### Univariate and multivariate analysis

After PSM, we examined prognostic factors for OS in all patients. In the univariate analysis, Gender (HR (95%CI): 0.627 (0.491–0.800), *P* < 0.001), peritoneal seeding (HR (95%CI): 2.830 (1.292–6.197), *P* = 0.020) and period of chemotherapy (HR (95%CI): 0.263 (0.143–0.484), *P* < 0.001) were significant predictors of overall survival. Then, multivariate analysis demonstrated that only peritoneal seeding (HR (95%CI): 3.308 (1.426–7.674), *P* = 0.014) and period of chemotherapy (HR (95%CI): 0.226 (0.139–0.508), *P* < 0.001) remained as prognostic factors (Table. 2).

**Table 2:** Univariate and multivariate analysis of overall survival in gastric cancer patients with outlet obstruction after propensity score matching

Variables	Univariate analysis		Multivariate analysis	
	HR (95%CI)	*P* value	HR (95%CI)	*P* value
Age (years)		0.856		
≤60	1			
> 60	0.958 (0.604–1.519)			
Gender		**< 0.001**		0.099
Male	1		1	
Female	0.627 (0.491–0.800)		0.646 (0.384–1.086)	
PS		0.057		
0/1	1			
2/3	1.587 (0.986–2.555)	0.139		
GOOSS		0.125		
0	1			
1	0.661 (0.389–1.122)			
CEA		0.067		
< 5 ng/ml	1			
≥5 ng/ml	1.557 (0.969–2.503)			
Ascites		0.122		
No	1			
Yes	1.459 (0.904–2.355)			
Invasion of adjacent organs		0.181		
No	1			
Yes	1.464 (0.838–2.558)			
Peritoneal seeding		**0.020**		0.014
P0	1		1	
P1/2	1.781 (0.816–3.884)	0.147	2.087 (0.923–4.719)	0.077
P3	2.830 (1.292–6.197)	0.009	3.308 (1.426–7.674)	0.005
Period of chemotherapy		**< 0.001**		< 0.001
0	1		1	
1–4	0.464 (0.269–0.803)	0.006	0.405 (0.226–0.727)	0.002
≥5	0.263 (0.143–0.484)	< 0.001	0.226 (0.139–0.508)	< 0.001
Treatment		0.245		
Gastrojejunostomy group	1			
Palliative gastrectomy group	1.315 (0.829–2.084)			

*HR* hazard ratio, *CI* confidence interval

### Postoperative outcomes

Table.3 shows postoperative surgical outcome data of these two procedures. In the gastrojejunostomy group, postoperative complications included three cases of abdominal bleeding, three cases of abdominal infection, two cases of pneumonia, one case of kidney failure and one case of stroke. In the palliative gastrectomy group, postoperative complications included two cases of abdominal bleeding, three cases of abdominal infection, two cases of incomplete ileus and two cases of pneumonia. The overall postoperative complication rates in the gastrojejunostomy group and palliative gastrectomy group were 19.23% (10/52) and 17.31% (9/52), respectively (*p* = 0.800), and no treatment-related death was observed in both groups (*p* = 1.000).

**Table 3:** Postoperative outcomes of gastric cancer patients with outlet obstruction

	Gastrojejunostomy group (*n* = 52)	Palliative gastrectomy group (*n* = 52)	*P* value
Postoperative complications			
Overall	10	9	0.800
Abdominal bleeding	3	2	
Abdominal infection	3	3	
Incomplete ileus	0	2	
Pneumonia	2	2	
Kidney failure	1	0	
Stroke	1	0	
Time of first oral intake (days)^a^	5.82 (4–11)	6.14 (4–15)	0.405
Postoperative hospital stay (days)^a^	10.84 (4–22)	14.37 (7–53)	0.007
Hospital death	0	0	1.000

^a^ Data are shown as mean (range)

The time of first oral intake was 5.82 ± 1.58 days (95%CI: 5.44–6.28 days) in the gastrojejunostomy group and 6.14 ± 2.14 days (95%CI: 5.62–6.81 days) in the palliative gastrectomy group, there was no significant difference in the time of first oral intake between the two groups (*P* = 0.405). However, the postoperative hospital stay was shorter in the gastrojejunostomy group than the palliative gastrectomy group (10.84 ± 3.61 days (95%CI: 9.77–11.83 days) vs. 14.37 ± 8.26 days (95%CI: 12.11–16.74 days), *P* = 0.007).

## Discussion

Gastric cancer with outlet obstruction is a life-threatening condition and usually associated with a dismal prognosis. Treatment selection was frequently done depending on the patient’s condition and willing in the real clinical practice. In this study, we focused on the value of palliative surgical resection for advanced gastric cancer with outlet obstruction.

Recently, the value of palliative surgery for patients with gastric cancer has been evaluated in some prospective trials. The GYMSSA trial was aimed to compare palliative gastrectomy plus chemotherapy versus chemotherapy alone for patients with metastatic gastric cancer, but was closed prematurely due to poor participant accrual. Finally, only 17 patients were enrolled, therefore, it was difficult to draw any conclusions [15]. The REGATTA trial revealed that gastrectomy followed by chemotherapy did not show any survival benefit compared with chemotherapy alone in advanced gastric cancer with a single non-curable factor. However, its subgroup analysis indicated that the distal advanced gastric cancer may still benefit from gastrectomy [8]. And most of the patients with GOO were distal advanced gastric cancer. Only a handful of retrospective studies have focused on this point. Keränen I et al. [6] compared three palliative methods to treat gastric outlet obstruction and found that palliative resection seems to provide a survival benefit in contrast with endoscopic stenting and gastrojejunostomy. In 2014, Okumura Y et al. [10] performed a retrospective analysis and showed that palliative distal gastrectomy offers no survival benefit as compared to gastrojejunostomy. However, the sample size was only 43 cases, indicating the low power to make this conclusion. These studies did not address risk adjustment; therefore, a selection bias may be present between palliative gastrectomy and gastrojejunostomy. In the present study, palliative gastrectomy was performed in better PS, lower CEA, less ascites, less invasion of adjacent organs and less peritoneal seeding patient. These patients had less tumor burden, more tolerance to chemotherapy and finally lead to better overall survival [16–20]. Consequently, the survival difference before PSM may be account for selection bias. After PSM, the obtained results revealed no significant differences in OS between these two therapies.

**Fig. 2:**
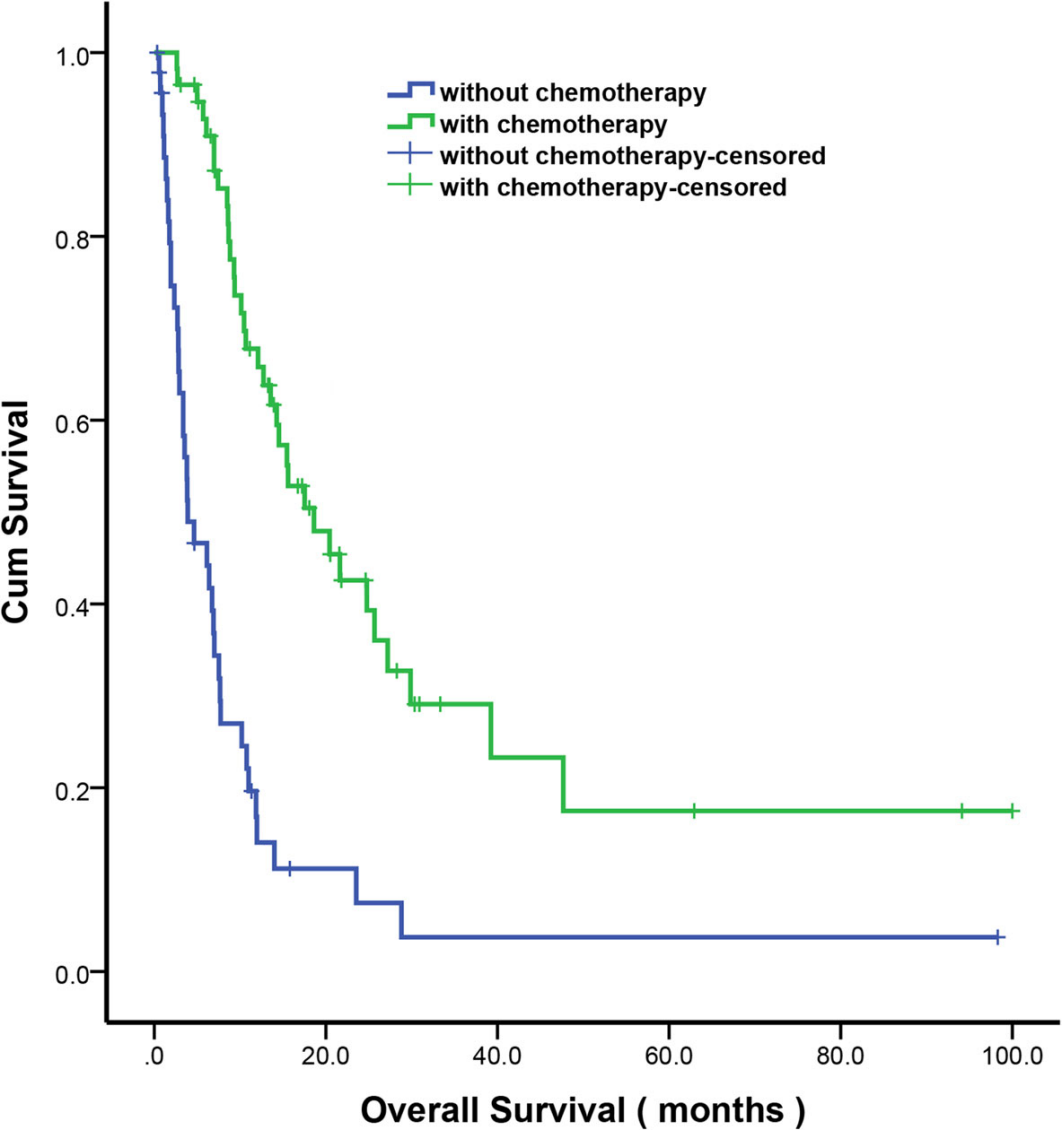
Kaplan-Meier survival curves of gastric cancer patients with outlet obstruction according to chemotherapy after propensity score matching (*P* < 0.001). *P*-values were calculated using the log-rank test

The present study demonstrated that the palliative gastrectomy or gastrojejunostomy was not an independent risk factor for survival in the multivariate analysis, whereas the peritoneal seeding and postoperative chemotherapy were independent prognostic factors for survival. Among the metastasis patterns of gastric cancer, peritoneal seeding is the most frequent pattern and cause of death in patients with gastric cancer [21]. In our study, 81.4% of patients were diagnosed with peritoneal seeding at the time of diagnosis. For gastric cancer patient with peritoneal seeding, several investigators have suggested that palliative gastrectomy should be recommended in selective P1 patients with peritoneal implantation [12, 20]. However, peritoneal seeding is often more severe in patients with GOO. In our study, 60.3% patients suffered from P2/3 peritoneal implantation, no studies have been conducted to verify this in patients with GOO. At present, a lot of prospective studies have defined chemotherapy as a standard of care for stage IV gastric cancer [3, 22–24]. In the present study, after propensity score matching, the median OS of patients who underwent chemotherapy was significantly better than who not (17.53 m versus. 6.13 m; *P* < 0.001) (Fig. 2), in agreement with other studies [25, 26].

In our study, the overall postoperative morbidity in the gastrojejunostomy group and palliative gastrectomy group were 19.23% (10/52) and 17.31% (9/52), respectively, and no treatment-related death was observed in patients who underwent palliative gastrectomy. Previous studies reported that the morbidity of palliative gastric resection ranged from 10.4 to 42% [12, 27, 28]. Hence, we considered that palliative gastrectomy was a safe procedure for patients with GOO. Nevertheless, the postoperative hospital stay was longer in the palliative gastrectomy group than the gastrojejunostomy group, which may lead to higher medical costs. As a whole, palliative gastrectomy cannot improve the prognosis, and the potential risk is theoretically high, therefore, the gastrojejunostomy is sufficient for patients with GOO.

There are some limitations in our study. First, this study is a retrospective study, and the data collection was in single center. Second, because of the rarity of gastric outlet obstruction due to gastric cancer, the study time span was over 15 years and many treatments are changing, especially in chemotherapy. Third, the quality of life after surgery is also an important concern for patients with GOO, but that was not been well evaluated in the present study. However, the number of patients enrolled in our study was relatively large compared to previous studies. Additionally, we used propensity score matching to balance the selection bias and explore the value of palliative gastrectomy for advanced gastric cancer with outlet obstruction. In the future, prospective studies are needed to confirm the results.

## Conclusion

The present study indicated that although the surgical complications of palliative gastrectomy were manageable, it showed no survival benefit. Therefore, relieving obstruction symptoms, improving patients’ quality of life, and creating better conditions for chemotherapy appear to be the main therapeutic strategies for advanced gastric cancer patients with GOO.

## Data Availability

The data used to support the findings of this study are included within the article.

## Abbreviations

GOO: Gastric outlet obstruction
PSM: Propensity Score Matching Analysis
OS: Overall survival
ECOG PS: Eastern Cooperative Oncology Group performance score
GOOSS: Gastric outlet obstruction scoring system
CEA: Carcinoembryonic antigen
CI: Confidence intervals
HR: hazard ratio
